# Trajectory Tracking Coordinated Control of 4WID-4WIS Electric Vehicle Considering Energy Consumption Economy Based on Pose Sensors

**DOI:** 10.3390/s23125496

**Published:** 2023-06-11

**Authors:** Yiran Qiao, Xinbo Chen, Zhen Liu

**Affiliations:** School of Automotive Studies, Tongji University, Shanghai 201804, China

**Keywords:** 4WID-4WIS EVs, trajectory tracking control, multi-objective coordinated control, mutant particle swarm optimization (MPSO)

## Abstract

In order to improve the stability and economy of 4WID-4WIS (four-wheel independent drive—four-wheel independent steering) electric vehicles in trajectory tracking, this paper proposes a trajectory tracking coordinated control strategy considering energy consumption economy. First, a hierarchical chassis coordinated control architecture is designed, which includes target planning layer, and coordinated control layer. Then, the trajectory tracking control is decoupled based on the decentralized control structure. Expert PID and Model Predictive Control (MPC) are employed to realize longitudinal velocity tracking and lateral path tracking, respectively, which calculate generalized forces and moments. In addition, with the objective of optimal overall efficiency, the optimal torque distribution for each wheel is achieved using the Mutant Particle Swarm Optimization (MPSO) algorithm. Additionally, the modified Ackermann theory is used to distribute wheel angles. Finally, the control strategy is simulated and verified using Simulink. Comparing the control results of the average distribution strategy and the wheel load distribution strategy, it can be concluded that the proposed coordinated control not only provides good trajectory tracking but also greatly improves the overall efficiency of the motor operating points, which enhances the energy economy and realizes the multi-objective coordinated control of the chassis.

## 1. Introduction

4WID-4WIS EV is a novel electric vehicle that reduces mechanical components, such as differentials and half shafts. It also integrates four-wheel steering technology based on a distributed four-wheel drive system. This allows four wheels to be driven and steered independently, increasing the controllable degree of freedom. In a word, 4WID-4WIS EV has unique advantages in vehicle dynamics control [1,2].

To fully exploit the control potential of 4WID-4WIS EV and improve the overall performance of the vehicle, coordinated multi-objective control of the chassis has become a current research focus [3,4,5]. In particular, stability and economy are two important performance characteristics. It is difficult to improve one performance by controlling only one performance, and in extreme cases, the effect deteriorates. Therefore, there is a need for coordinated control of the two performances to make the vehicle stable and economical at the same time, which has been researched and studied by several scholars [6]. In [7], a 4WID EV torque coordination control strategy is developed, which considers both stability and economy and uses MPC to calculate the generalized force. The demand torque of each wheel is determined by an online solution using the control distribution error, tire utilization rate, and power consumption of the drive system as objective functions. In [8], a multi-objective online optimization of energy management strategy for 4WID EV is proposed. It considers the efficiency of the drive system, tire slip energy consumption, wheel torque fluctuation, yaw rate tracking error, etc. The weights of each element are dynamically adjusted using the fuzzy control method, and the effectiveness of the strategy is verified by simulations. In [9], a cooperative game-based actuator fault-tolerant control strategy for a 4WID EV is proposed. This strategy minimizes tire energy dissipation to ensure economic efficiency by designing a two-dimensional game controller that simultaneously satisfies the generalized forces required for vehicle stability.

Due to the great potential of intelligent driving technology to enhance vehicle safety [10,11], improve traffic efficiency, and reduce energy consumption [12], the research on trajectory tracking control of 4WID-4WIS electric vehicles has received increasing attention in the automotive industry, and numerous control methods have been developed [13,14,15]. In [16], LTV-MPC (Linear Time-Varying Model Predictive Control) based on DYC (Direct Yaw Control) is used to realize velocity tracking and trajectory tracking, which improves stability during trajectory tracking. In [17], a robust path-tracking controller for 4WID-4WIS agricultural robotic vehicles is designed by combining the backstepping SMC (Sliding Mode Control) theory to improve the robustness of trajectory tracking. In [18], a four-wheel steering controller and a velocity tracking controller for 4WID-4WIS EV are designed based on the SMC algorithm to improve the accuracy of trajectory tracking. In [19], the feedback linear quadratic regulator (LQR) controller is used to realize emergency avoidance under 4WS high-speed conditions.

There are certain shortcomings in the aforementioned research which can be summarized as follows. Most studies on chassis coordinated control is related to traditional 4WID EV, while there is relatively little research on 4WID-4WIS EV. In addition, most existing research establishes multi-objective optimization problems and solves them online to obtain the control variables. This not only makes the objective function too complex and difficult to solve but also has a negative impact on the real-time performance of the controller. Last but not least, many studies on the trajectory tracking control of 4WID-4WIS EV mostly only consider the trajectory tracking performance. Some studies also consider stability, but there is almost no research that simultaneously considers trajectory tracking performance, stability and economy.

To improve the stability and economy of 4WID-4WIS EV in trajectory tracking, the contributions of this paper are summarized as follows:

- A hierarchical architecture for chassis coordinated control is designed including target planning layer, and coordinated control layer.
- The MPC path tracking controller is constructed, which takes into account stability constraints, such as yaw rate and tire slip angle.
- The MPSO algorithm is also used to create a mapping of the distribution coefficients by optimizing the torque distribution between the front and rear wheels offline, thereby reducing the computational cost.

The paper is organized as follows: Section 2 proposes a hierarchical chassis coordination control. Section 3 highlights the simulation validation and compares the simulation results of different torque distribution strategies. Section 4 sums up the conclusions.

## 2. Trajectory Tracking Coordinated Control

### 2.1. Vehicle Model

4WID-4WIS EV uses in-wheel motors and steering motors to replace the traditional transmission and steering mechanism, giving it more control freedom and thus enabling more advanced control methods to improve the overall performance of the vehicle. Considering the strong coupling between the vehicle subsystems and the strong nonlinearity of the tires and motor actuators, it is of great significance to establish a reasonable and accurate vehicle model to study the control strategy of 4WID-4WIS EV.

#### 2.1.1. Vehicle Dynamic Model

The vehicle is a complex multi-body dynamic system, and the vehicle dynamic model is the basis for dynamic analysis, active control, function realization, and parameter optimization. According to the research objective and concern, many scholars have proposed various linear and nonlinear vehicle models with different degrees of complexity [20,21]. This work focuses on the longitudinal and lateral coupling control of the 4WID-4WIS EV. The coupling effects between longitudinal motion and lateral motion of the vehicle include three categories: kinematic coupling, tire force coupling, and load transfer coupling. Kinematic coupling refers to the longitudinal motion being influenced by the longitudinal component of the steering wheel lateral deflection force due to the presence of the wheel steering angle. Additionally, the lateral motion is also influenced by the longitudinal velocity. Tire force coupling is the interaction between tire lateral and longitudinal forces, the combined force of which is constrained by the friction ellipse. The vertical load redistribution is caused by longitudinal acceleration or lateral acceleration, which in turn influences the longitudinal and lateral dynamics. Therefore, a vehicle dynamic model with seven degrees of freedom is adopted, i.e., longitudinal motion, lateral motion, yaw motion, and the rotation of four wheels.

**Figure 1:**
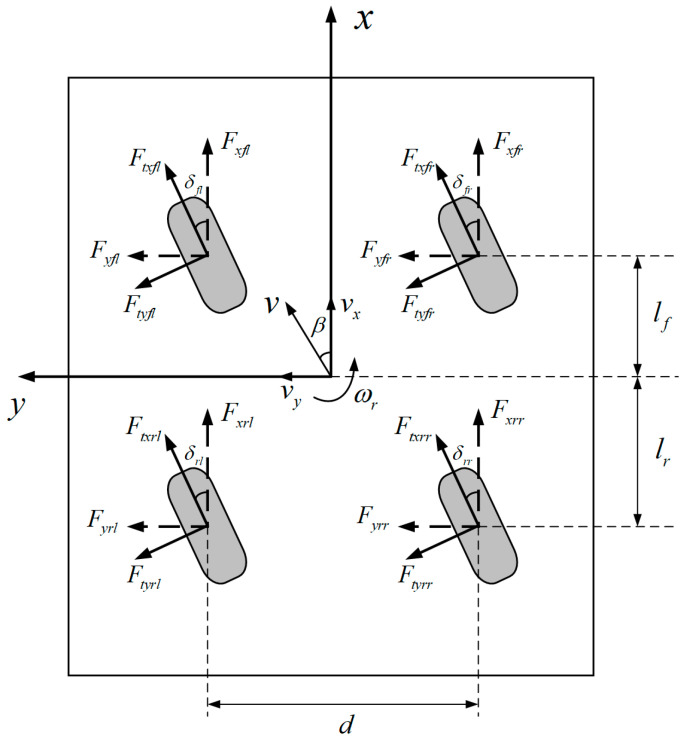
4WID-4WIS vehicle dynamic model.

The vehicle is assumed to be left-right symmetric about the center plane and driven on a flat horizontal road, ignoring the vertical motion of the body. At the same time, it is assumed that the suspension system is a rigid structure and the body pitch and roll motion is neglected. After conducting the above modifications, the simplified dynamic model is developed, as shown in Figure 1.

The longitudinal motion equation is:

(1) $$m\left({\dot{v}}_{x}-v_{y}\omega_{r}\right)=F_{xfl}+F_{xfr}+F_{xrl}+F_{xrr}$$

The lateral motion equation is:

(2) $$m\left({\dot{v}}_{y}+v_{x}\omega_{r}\right)=F_{yfl}+F_{yfr}+F_{yrl}+F_{yrr}$$

The equation of yaw motion is:

(3) $$I_{z}{\dot{\omega }}_{r}=\left(F_{xfr}+F_{xrr}-F_{xfl}-F_{xrl}\right)\frac{d}{2}+\left(F_{yfl}+F_{yfr}\right)l_{f}-\left(F_{yrl}+F_{yrr}\right)l_{r}$$

where,

(4) $$F_{xij}=F_{txij}\cos\delta_{ij}-F_{tyij}\sin\delta_{ij}$$

(5) $$F_{yij}=F_{txij}\sin\delta_{ij}+F_{tyij}\cos\delta_{ij}$$

(6) $$i\in \left\{f,r\right\},\;j\in \left\{l,r\right\}$$

The equation of wheel motion is:

(7) $$I_{w}{\dot{\omega }}_{ij}=T_{dij}-F_{txij}R_{eff}-T_{bij}$$

where, m is the vehicle mass, $v_{x}$ is the longitudinal velocity, $v_{y}$ is the lateral velocity, $\omega_{r}$ is the yaw rate, $F_{xij}$ and $F_{yij}$ are the longitudinal force and lateral force of each wheel, respectively, $I_{z}$ is the yaw inertia, $l_{f}$ is the distance from the center of mass to the front axle, $l_{r}$ is the distance from the center of mass to the rear axle, d is the wheelbase of the vehicle, $F_{txij}$ and $F_{tyij}$ are the longitudinal and lateral forces of each wheel in the tire coordinate system, respectively, $\delta_{ij}$ is the wheel angle, $I_{w}$ is the wheel moment of inertia, $\omega_{ij}$ is the speed of each wheel, $T_{dij}$ is the driving torque of each wheel, $T_{bij}$ is the braking torque of each wheel, and $R_{eff}$ is the effective rolling radius of the wheel.

#### 2.1.2. Tire Model

As the only component that connects the vehicle to the ground, any state of vehicle motion depends on the interaction forces between the tire and the road surface. The tire has a critical impact on the vehicle’s performance, so it is necessary to perform accurate dynamic modeling of tires to better describe the effects of tire forces on vehicle dynamics. The tire model describes the relationship between the tire motion parameters and tire forces, which can be mainly divided into three types: theoretical model, empirical model, and semi-empirical model [22,23]. The theoretical model is formed by studying the deformation mechanism of tires based on the physical essence of tire mechanics, but its complex structure is not conducive to simulation research. The empirical model is based on the experimental tire data, but as a result, it also lacks theoretical support and is poorly scalable. The semi-empirical model combines the advantages of both models with theoretical support and experimental data to obtain key parameters that ensure good accuracy and scalability.

In this study, the most commonly used semi-empirical tire model MF (Magic Formula) is selected. The combination formula of trigonometric functions is used to describe the relationship between tire force, slip rate, and slip angle. This model is suitable for the operating condition with combined longitudinal and lateral forces. The general expression of the MF tire model is as follows:

(8) $$\left\{\begin{array}{l} y=D\sin\left\{C\arctan\left[Bx-E\left(Bx-\arctan\left(Bx\right)\right)\right]\right\}\\ Y\left(X\right)=y\left(x\right)+S_{V}\\ x=X+S_{H} \end{array}\right.$$

where, B is the stiffness factor, C is the shape factor, D is the peak factor, E is the curvature factor, Y is the output variable, that is, longitudinal force, lateral force or aligning torque, X is the input variable, that is, slip rate or slip angle, $S_{V}$ is the vertical offset, and $S_{H}$ is the horizontal offset.

As input variables for the MF tire model, slip rate and side slip angle are important parameters for calculating the tire effect, which must be calculated from the vehicle state as shown in Equations (9) and (10), respectively.

(9) $$s_{ij}=\left\{\begin{array}{l} \begin{array}{ll} \frac{\omega_{ij}R_{eff}-u_{ij}}{\omega_{ij}R_{eff}} & \omega_{ij}R_{eff}\geq u_{ij}\left(Drive\right) \end{array}\\ \begin{array}{ll} \frac{\omega_{ij}R_{eff}-u_{ij}}{u_{ij}} & \omega_{ij}R_{eff}<u_{ij}\left(Brake\right) \end{array} \end{array}\right.$$

where, $u_{ij}$ is the speed of the wheel center.

(10) $$\left\{\begin{array}{c} \alpha_{fl}=\tan^{-1}\left(\frac{v_{y}+\omega_{r}l_{f}}{v_{x}-\omega_{r}\cdot d/2}\right)-\delta_{fl}\\ \alpha_{fr}=\tan^{-1}\left(\frac{v_{y}+\omega_{r}l_{f}}{v_{x}+\omega_{r}\cdot d/2}\right)-\delta_{fr}\\ \alpha_{rl}=\tan^{-1}\left(\frac{v_{y}-\omega_{r}l_{r}}{v_{x}-\omega_{r}\cdot d/2}\right)-\delta_{rl}\\ \alpha_{rr}=\tan^{-1}\left(\frac{v_{y}-\omega_{r}l_{r}}{v_{x}+\omega_{r}\cdot d/2}\right)-\delta_{rr} \end{array}\right.$$

#### 2.1.3. Motor Model

The 4WID-4WIS EV drive system consists of four in-wheel motors, and independent four-wheel steering is achieved by four steering motors. Therefore, to investigate the longitudinal and lateral coordinated control strategy, the characteristics of the motors are first analyzed, and suitable motor models are established.

1. In-wheel motor model

The research focuses on the coordinated control of the vehicle with multiple objectives. For the in-wheel motor model, the charging and discharging efficiency of the motor is only for the steady-state characteristics. The transient characteristics of the motor are not considered. Therefore, the charging and discharging efficiency of the motor can be expressed as in Equation (11).

(11) $$\eta_{em}=\eta \left(n_{em},T_{em}\right)$$

where, $n_{em}$ is the motor speed, and $T_{em}$ is the motor torque.

**Figure 2:**
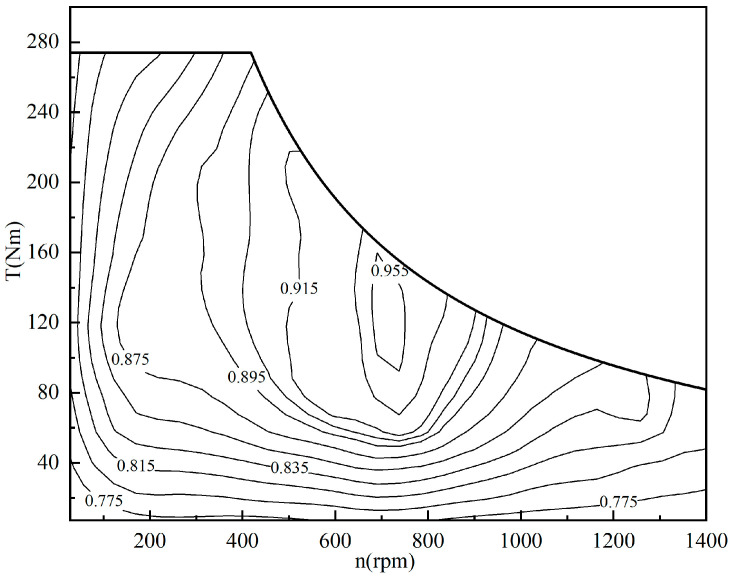
In-wheel motor efficiency map.

Since the driving state of the vehicle is mainly studied in this paper, the motor efficiency characteristics are shown in Figure 2. The external characteristic curve and the efficiency map are used to characterize the in-wheel motor. The external characteristic curve can be used to determine the maximum torque as a function of wheel speed in real-time. The discharge efficiency of the motor can be determined using a two-dimensional look-up table of motor speed and torque.

2. Steering Motor Model

Since the steering motor in this study is not focused on the efficiency characteristics, it is simplified from the perspective of the motor actuation effect as a wheel angle tracking model. The delay caused by the steering mechanism is considered as shown in Equation (12).

(12) $$\delta_{ij\_out}=\delta_{ij\_req}\cdot \frac{1}{1+\tau s}$$

where, $\delta_{ij\_out}$ is the actual output angle, $\delta_{ij\_req}$ is the required output angle, and τ is the response time constant.

### 2.2. Vehicle State Acquisition

Before performing chassis coordination control, it is necessary to observe the lateral and longitudinal states of the vehicle, including the vehicle’s position information and the body’s pose information. The position information of the vehicle consists of the coordinates of the vehicle’s lateral and longitudinal axes, velocity and acceleration information, and the pose information of the body includes yaw rate, sides lip angle, pitch angle, roll angle and so on.

The implementation of the control strategy is based on the acquisition of vehicle state information by pose sensors. There have been many studies on how to obtain the pose information of the vehicle. For example, RTK (Real Time Kinematic) and INS (Inertial Navigation System) are combined for positioning. Body combination sensors, including longitudinal acceleration sensors, lateral acceleration sensors and yaw rate sensors are used for pose calculation.

Considering that the signals from pose sensors contain uncertain noise interference, it is necessary to design a reasonable signal filter to obtain accurate pose signals. At the same time, the acquisition of road condition information, such as road adhesion coefficient and road slope, can be realized by designing corresponding state observers based on the information from pose sensors. Since the focus of this paper is on the trajectory tracking coordinated control, this part will not be elaborated in detail.

### 2.3. Chassis Control Architecture

Based on the vehicle states obtained by pose sensors, the trajectory tracking controller receives the expected path and velocity information sent by the decision planning layer and controls the longitudinal and lateral movement of the vehicle so that the vehicle follows the expected path. In addition, 4WID-4WIS EV has multiple degrees of freedom, and the driving and steering of each wheel are controllable, which provides a basis for realizing multi-objective coordinated control of the chassis. In summary, this research aims to achieve the expected trajectory tracking while improving driving stability and energy economy.

**Figure 3:**
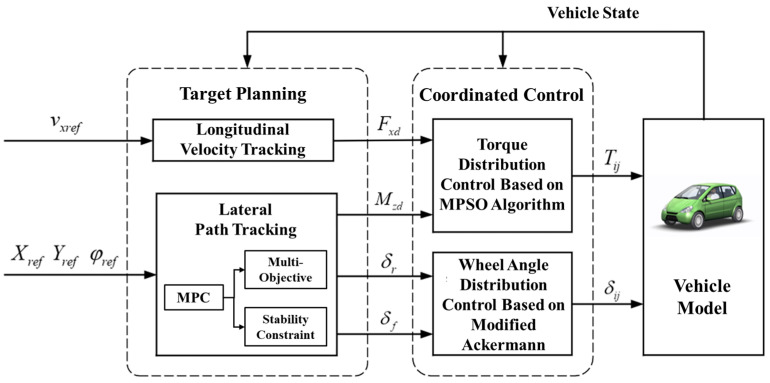
The chassis coordinated control architecture.

The chassis control architecture designed in this paper is shown in Figure 3, which has a layered structure and is divided into target planning layer and coordinated control layer.

Based on the decentralized control structure, the target planning layer receives the expected path and velocity information and decouples the longitudinal and lateral control targets. In the longitudinal direction, an expert PID control method is used to track the desired velocity and output the generalized longitudinal force. In the lateral direction, the MPC algorithm is used to perform the multi-objective real-time rolling optimization considering the stability constraints, such as yaw rate and tire slip angle. It outputs the generalized yaw moment and the generalized wheel angle.

The coordinated control layer distributes the torque and wheel angle of each wheel according to the generalized force, generalized yaw moment, and generalized wheel angle, which realizes the calculation of the control objective of the motor actuator. Considering the efficiency characteristics of the in-wheel motor, the torque distribution control uses the MPSO algorithm to optimize the torque of the front and rear wheels offline. Then, the two-dimensional diagram of the optimal torque distribution coefficient is generated and real-time table look-up is performed according to the vehicle state to calculate the torque for each wheel. The wheel angle distribution strategy considers the wheel slip angle and distributes the generalized wheel angle based on the modified Ackerman theory to obtain the wheel angles. Finally, the torque and angle of each wheel are output to in-wheel motors and steering motors to realize the closed-loop control of the strategy.

### 2.4. Target Planning

The target planning layer completes the resolution of the chassis control targets based on the expected trajectory. The expected vehicle trajectory includes information about the expected path and velocity, i.e., the expected coupling of the vehicle’s longitudinal and lateral motion states. Trajectory tracking strategies can be divided into decentralized control and centralized control according to different control structures. Decentralized control [24,25] refers to the decoupling of longitudinal and lateral vehicle motion, i.e., it decomposes the trajectory tracking problem into longitudinal velocity tracking and lateral path tracking problems and designs corresponding control objectives. Centralized control [26,27,28], on the other hand, means that the longitudinal and lateral motion in the trajectory tracking problem is considered uniformly, with a global controller computing the longitudinal and lateral control targets. The centralized trajectory tracking controller considers the system holistically and can better account for the coupling properties between longitudinal and lateral motion control. However, the higher dimensionality and complexity of the system model make it more challenging to design control laws than decentralized control, and it is also more computationally intensive and costly to implement physically. In contrast, the decentralized trajectory tracking strategy is relatively simple and easy to implement in engineering practice. In this work, the trajectory tracking strategy adopts a decentralized structure, decouples the trajectory tracking problem into a longitudinal velocity tracking problem and a lateral path tracking problem, and designs the tracking strategy to calculate the desired generalized force and moment.

#### 2.4.1. Longitudinal Velocity Tracking

The uncertainty of vehicle system parameters, uncertain external disturbances, and nonlinear coupling between systems lead to difficulties in tuning PID control parameters and low robustness of dynamic and steady-state performance of vehicle velocity tracking [29]. In this work, the expert PID algorithm is used in conjunction with the expert system theory to track the longitudinal velocity.

The expert PID algorithm is based on various knowledge of the controlled object and the control laws. If the precise model of the controlled object is not available, the PID parameters are designed using expert experience. The deviations and increments of deviations are used to determine the current state of the controlled system. Rules are designed to adjust the output of the controller to achieve faster and smoother convergence of the controlled system.

Assume that $e(k)=v(k)-v_{d}(k)$ is the velocity error, and Δe(k)=e(k)−e(k−1) is the velocity error increment. At the same time, based on engineering practice experience, the maximum deviation value is set as $M_{max}$, the middle deviation value is set as $M_{mid}$ and the minimum deviation value is set as $M_{\min}$. According to the setting of deviation, deviation increment and extreme value of deviation, the setting rules are as follows:

(1) When $\left|e\left(k\right)\right|>M_{max}$, it means that the velocity error is unacceptably large. At this time, the controller should be directly output at full load, that is, $u(k)=F_{\mathrm{xmax}}$.
(2) When $e\left(k\right)*\Delta e\left(k\right)>0,\;\Delta e\left(k\right)=0$, it means that the velocity deviation is changing in the direction of increasing the absolute value of the deviation, or the deviation is a certain fixed value, then
  (13) $$u(k)=u(k-1)+k_{1}\left\{k_{i}e(k)+k_{p}\Delta e(k)+k_{d}\Delta \Delta e(k)\right\}$$

If $\left|e\left(k\right)\right|>M_{mid}$, the velocity deviation is also large, and it may be considered to increase the output gain $k_{1}$ of the controller.

(3) When e(k)Δe(k)<0, Δe(k)Δe(k−1)>0,e(k)=0, it means that the absolute value of the velocity deviation is changing in the direction of decreasing, or has reached the equilibrium state. Then, the controller output remains unchanged, that is, $u\left(k\right)=u\left(k-1\right)$.
(4) When e(k)Δe(k)<0, Δe(k)Δe(k−1)<0, it means that the velocity deviation is in the limit state, then,
  (14) $$u(k)=u(k-1)+k_{2}k_{i}e(k)$$

If $\left|e\left(k\right)\right|>M_{mid}$, it means that the velocity deviation is also large, and it may be considered to increase the output gain $k_{2}$ of the controller.

(5) When $\left|e\left(k\right)\right|<M_{min}$, it means that the absolute value of the velocity deviation is very small. In order to reduce the static error of the system, PI control is implemented:
  (15) $$u(k)=u(k-1)+k_{i}e(k)+k_{p}\Delta e(k)$$

To sum up, the generalized longitudinal force is expressed as:

(16) $$F_{xd}(k)=u(k)$$

#### 2.4.2. Lateral Path Tracking

Intelligent driving faces a complex and changing environment where conditions, such as critical safety constraints and actuator constraints, must be met [30,31,32]. In this paper, MPC is used to realize the lateral path tracking considering the driving stability constraints.

1. Predictive model design

**Figure 4:**
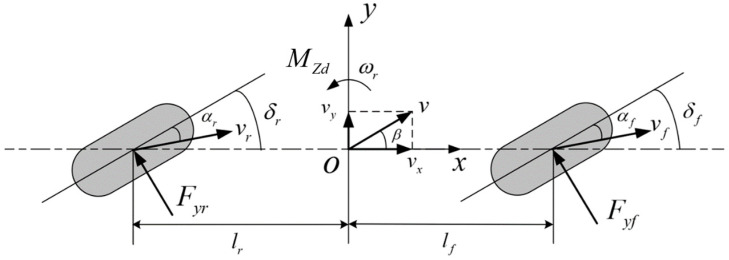
Nonlinear three degrees-of-freedom vehicle model.

The predictive model is the basis of the MPC used to predict the future output of the controlled system. The vehicle is a complex coupled nonlinear dynamic model. More degrees of freedom can improve the modeling accuracy, but the complexity of the model will also increase, making it difficult to meet the requirements for fast model solutions and real-time control. Therefore, dynamic modeling must strike a balance between improving model accuracy and reducing model complexity. Considering the accuracy and real-time requirements for lateral control of vehicles, a nonlinear three degrees-of-freedom vehicle model with lateral and longitudinal coupling is established in this paper to realize the prediction of system output as shown in Figure 4.

Based on the established dynamic model, the force analysis is performed:

(17) $$\left\{\begin{array}{l} \begin{array}{l} m\left({\dot{v}}_{y}+v_{x}\omega_{r}\right)=C_{f}\left(\frac{v_{y}+l_{f}\omega_{r}}{v_{x}}-\delta_{f}\right)+C_{r}\left(\frac{v_{y}-l_{r}\omega_{r}}{v_{x}}-\delta_{r}\right)-mv_{x}\omega_{r}\\ I_{z}{\dot{\omega }}_{r}=l_{f}C_{f}\left(\frac{v_{y}+l_{f}\omega_{r}}{v_{x}}-\delta_{f}\right)-l_{r}C_{r}\left(\frac{v_{y}-l_{r}\omega_{r}}{v_{x}}-\delta_{r}\right)+M_{zd} \end{array}\\ \dot{\varphi }=\omega_{r}\\ \dot{x}=v_{x}\cos\varphi -v_{y}\sin\varphi \\ \dot{y}=v_{x}\sin\varphi +v_{y}\cos\varphi \end{array}\right.$$

where, φ is the heading angle, $C_{f}$ and $C_{r}$ are the equivalent cornering stiffnesses of the front axle and the rear axle, respectively, $\delta_{f}$ is the front wheel angle, $\delta_{r}$ is the rear wheel angle, $M_{zd}$ is the generalized yaw moment, x and y are the longitudinal and lateral coordinates of the centroid in the geodetic coordinate system, respectively.

In the path tracking process, the desired heading angle and lateral coordinate can be obtained from the longitudinal coordinate of the current vehicle position. The state variable X, the control variable u and the output variable Y of the controller can be shown as:

(18) $$\begin{array}{l} X={\left[v_{y},\varphi ,\omega_{r},y,x\right]}^{T}\\ u={\left[\delta_{f},\delta_{r},M_{zd}\right]}^{T}\\ Y={\left[\varphi ,y\right]}^{T} \end{array}$$

Assuming that the longitudinal velocity remains constant during path tracking, in order to meet the real-time requirements of high-speed control, this paper uses Taylor’s formula to linearize the nonlinear system. The Taylor expansion is performed at the reference point, and the high-order differential components are rounded off. Then, the discretization is carried out using the first-order difference quotient method, as shown in Equation (19).

(19) $$X\left(k+1\right)=aX\left(k\right)+bu\left(k-1\right)+b\Delta u\left(k\right)+d\left(k\right)$$

where,

(20) $$\begin{array}{l} a=I+T\frac{\partial f}{\partial X},\;b=T\frac{\partial f}{\partial u}\\ d\left(k\right)=X_{r}\left(k+1\right)-aX_{r}\left(k\right)-bu_{r}\left(k\right) \end{array}$$

the augmented state variables are constructed:

(21) $$\xi \left(k+1\right)=\left[\begin{array}{c} X\left(k+1\right)\\ u\left(k\right) \end{array}\right]=\left[\begin{array}{cc} a & b\\ 0_{N_{u}\times N_{x}} & I_{N_{u}\times N_{u}} \end{array}\right]\xi \left(k\right)+\left[\begin{array}{c} b\\ I_{N_{u}\times N_{u}} \end{array}\right]\Delta u\left(k\right)+\left[\begin{array}{c} d\left(k\right)\\ 0_{N_{u}\times 1} \end{array}\right]$$

where, $N_{x}$ is the number of state variables, and $N_{u}$ is the number of control variables. Equation (21) can be simplified as

(22) $$\xi \left(k+1\right)=A\xi \left(k\right)+B\Delta u\left(k\right)+\bar{d}\left(k\right)$$

where,

(23) $$A=\left[\begin{array}{cc} a & b\\ 0_{N_{u}\times N_{x}} & I_{N_{u}\times N_{u}} \end{array}\right],\;B=\left[\begin{array}{c} b\\ I_{N_{u}\times N_{u}} \end{array}\right],\;\bar{d}\left(k\right)=\left[\begin{array}{c} d\left(k\right)\\ 0_{N_{u}\times 1} \end{array}\right]$$

Therefore, the discretized output equation can be obtained as:

(24) $$Y\left(k+1\right)=C\xi \left(k+1\right)$$

where,

(25) $$C=\left[\begin{array}{cccccccc} 0 & 1 & 0 & 0 & 0 & 0 & 0 & 0\\ 0 & 0 & 0 & 1 & 0 & 0 & 0 & 0 \end{array}\right]$$

The prediction time-domain length is set as p, and the control time-domain length is set as c (p≥c). Assuming that the control increment outside the control time domain is zero, the predicted output equation of the system can eventually be obtained by iteration, as shown in Equation (26).

(26) $$Y_{p}\left(k\right)=\Phi \xi \left(k\right)+\Theta \Delta U\left(k\right)+\Gamma D\left(k\right)$$

where,

(27) $$Y_{p}\left(k\right)=\left[\begin{array}{l} Y\left(\left.k+1\right|k\right)\\ Y\left(\left.k+2\right|k\right)\\ Y\left(\left.k+3\right|k\right)\\ \begin{array}{c} \vdots \\ Y\left(\left.k+c\right|k\right)\\ \vdots \end{array}\\ Y\left(\left.k+p\right|k\right) \end{array}\right],\;\Delta U\left(k\right)=\left[\begin{array}{c} \Delta u\left(k\right)\\ \Delta u\left(k+1\right)\\ \Delta u\left(k+2\right)\\ \begin{array}{c} \vdots \\ \Delta u\left(k+c-1\right) \end{array} \end{array}\right],\;D\left(k\right)=\left[\begin{array}{c} \bar{d}\left(k\right)\\ \bar{d}\left(k+1\right)\\ \bar{d}\left(k+2\right)\\ \vdots \\ \bar{d}\left(k+c-1\right)\\ \vdots \\ \bar{d}\left(k+p-1\right) \end{array}\right]$$

(28) $$\Phi =\left[\begin{array}{l} CA\\ CA^{2}\\ CA^{3}\\ \begin{array}{c} \vdots \\ CA^{c}\\ \vdots \end{array}\\ CA^{P} \end{array}\right],\;\Theta =\left[\begin{array}{ccccc} CB & 0_{N_{y}\times N_{u}} & 0_{N_{y}\times N_{u}} & \cdots & 0_{N_{y}\times N_{u}}\\ CAB & CB & 0_{N_{y}\times N_{u}} & \cdots & 0_{N_{y}\times N_{u}}\\ CA^{2}B & CAB & CB & \cdots & 0_{N_{y}\times N_{u}}\\ \vdots & \vdots & \vdots & \vdots & \vdots \\ CA^{c-1}B & CA^{c-2}B & CA^{c-3}B & \cdots & CB\\ \vdots & \vdots & \vdots & \vdots & \vdots \\ CA^{p-1}B & CA^{p-2}B & CA^{p-3}B & \cdots & CA^{p-c}B \end{array}\right]$$

(29) $$\Gamma =\left[\begin{array}{ccccccc} C & 0_{N_{y}\times \left(N_{x}+N_{u}\right)} & 0_{N_{y}\times \left(N_{x}+N_{u}\right)} & \cdots & 0_{N_{y}\times \left(N_{x}+N_{u}\right)} & \cdots & 0_{N_{y}\times \left(N_{x}+N_{u}\right)}\\ CA & C & 0_{N_{y}\times \left(N_{x}+N_{u}\right)} & \cdots & 0_{N_{y}\times \left(N_{x}+N_{u}\right)} & \cdots & 0_{N_{y}\times \left(N_{x}+N_{u}\right)}\\ CA^{2} & CA & C & \cdots & 0_{N_{y}\times \left(N_{x}+N_{u}\right)} & \cdots & 0_{N_{y}\times \left(N_{x}+N_{u}\right)}\\ \vdots & \vdots & \vdots & \vdots & \vdots & \vdots & \vdots \\ CA^{c-1} & CA^{c-2} & CA^{c-3} & \cdots & C & \cdots & 0_{N_{y}\times \left(N_{x}+N_{u}\right)}\\ \vdots & \vdots & \vdots & \vdots & \vdots & \vdots & \vdots \\ CA^{p-1} & CA^{p-2} & CA^{p-3} & \cdots & CA^{p-c} & \cdots & C \end{array}\right]$$

2. Objective function design

The control objective of lateral path tracking is to ensure that the vehicle tracks the desired path smoothly, accurately, and quickly. Therefore, the objective function in this paper is designed as shown in Equation (30).

(30) $$J={\left[Y_{p}\left(k\right)-Y_{ref}\right]}^{T}Q_{Q}\left[Y_{p}\left(k\right)-Y_{ref}\right]+\Delta U{\left(k\right)}^{T}R_{R}\Delta U\left(k\right)$$

where, $Q_{Q}=I_{p}⊗Q$, $R_{R}=I_{c}⊗R$, Q is the weight coefficient matrix of the output of the control system, R is the weight coefficient matrix of the control increment, and ⊗ represents the Kronecker product.

The first in the objective function represents the deviation of the heading angle and the deviation of the lateral displacement, which characterizes the accuracy of the vehicle in tracking the desired path. The second limits the control increment, which not only ensures the stability and continuity of the control but also takes into account the response capability of the actuator. Equation (30) can be simplified as,

(31) $$J={\left[E+\Theta \Delta U\left(k\right)\right]}^{T}Q\left[E+\Theta \Delta U\left(k\right)\right]+\Delta U{\left(k\right)}^{T}R\Delta U\left(k\right)$$

where,

(32) $$E=\Phi \xi \left(k\right)+\Gamma D\left(k\right)-Y_{ref}$$

Considering that *E* is a constant matrix at each sampling moment, it can be omitted. The final objective function in standard quadratic programming form is obtained, as shown in Equation (33).

(33) $$J=\Delta U{\left(k\right)}^{T}\left(\Theta^{T}Q\Theta +R\right)\Delta U\left(k\right)+2E^{T}Q\Theta \Delta U\left(k\right)$$

3. Constraints design

A major advantage of MPC over other control methods is that it handles constraints better. MPC is a rolling optimization process that can naturally incorporate constraints into the optimization problem. For the path-following controller in this paper, the constraints are mainly considered in the form of the constraint on the control increment, the constraint on the control set, and the stability constraints.

In order to make the control process more stable and improve the stability and comfort of the vehicle when tracking the desired path, the control increment should be constrained. Due to the constraints of the actuator, there are also constraints on the control variables, such as front wheel angle, rear wheel angle, and generalized yaw moment. So, in summary, the following can be said:

(34) $$U_{\min}\left(k\right)\leq U_{ct}\left(k-1\right)+A_{ct}*\Delta U\left(k\right)\leq U_{\max}\left(k\right)$$

where,

(35) $$U_{ct}\left(k-1\right)=\left[\begin{array}{c} u\left(k-1\right)\\ u\left(k-1\right)\\ \vdots \\ u\left(k-1\right) \end{array}\right],\;A_{ct}=\left[\begin{array}{cccc} 1 & 0 & \cdots & 0\\ 1 & 1 & \cdots & 0\\ \vdots & \vdots & \vdots & \vdots \\ 1 & 1 & 1 & 1 \end{array}\right]⊗I_{c}$$

To ensure that the vehicle has good stability while tracking the desired path, stability constraints must be imposed on the MPC optimization problem. The stability constraints generally start with the two parameters yaw rate and tire slip angle. Therefore, the constraint output variable is defined as $Y_{b}={\left[\omega_{r},\alpha_{r}\right]}^{T}$. The expression for the constraint output can be obtained, as shown in Equation (36).

(36) $$Y_{b}\left(k+1\right)=C_{b}\xi \left(k+1\right)$$

where,

(37) $$C_{b}=\left[\begin{array}{cccccccc} 0 & 0 & 1 & 0 & 0 & 0 & 0 & 0\\ \frac{1}{v_{x}} & 0 & -\frac{l_{r}}{v_{x}} & 0 & 0 & 0 & -1 & 0 \end{array}\right]$$

The predicted output equation for the stability constraint is shown in Equation (38).

(38) $$Y_{p,b}\left(k\right)=\Phi_{b}\xi \left(k\right)+\Theta_{b}\Delta U\left(k\right)+\Gamma_{b}D\left(k\right)$$

where,

(39) $$Y_{p,b}\left(k\right)=\left[\begin{array}{l} Y_{b}\left(\left.k+1\right|k\right)\\ Y_{b}\left(\left.k+2\right|k\right)\\ Y_{b}\left(\left.k+3\right|k\right)\\ \begin{array}{c} \vdots \\ Y_{b}\left(\left.k+c\right|k\right)\\ \vdots \end{array}\\ Y_{b}\left(\left.k+p\right|k\right) \end{array}\right],\;\Phi_{b}=\left[\begin{array}{l} C_{b}A\\ C_{b}A^{2}\\ C_{b}A^{3}\\ \begin{array}{c} \vdots \\ C_{b}A^{c}\\ \vdots \end{array}\\ C_{b}A^{P} \end{array}\right]$$

(40) $$\Theta_{b}=\left[\begin{array}{ccccc} C_{b}B & 0_{N_{y1}\times N_{u}} & 0_{N_{y1}\times N_{u}} & \cdots & 0_{N_{y1}\times N_{u}}\\ C_{b}AB & C_{b}B & 0_{N_{y1}\times N_{u}} & \cdots & 0_{N_{y1}\times N_{u}}\\ C_{b}A^{2}B & C_{b}AB & C_{b}B & \cdots & 0_{N_{y1}\times N_{u}}\\ \vdots & \vdots & \vdots & \vdots & \vdots \\ C_{b}A^{c-1}B & C_{b}A^{c-2}B & C_{b}A^{c-3}B & \cdots & C_{b}B\\ \vdots & \vdots & \vdots & \vdots & \vdots \\ C_{b}A^{p-1}B & C_{b}A^{p-2}B & C_{b}A^{p-3}B & \cdots & C_{b}A^{p-c}B \end{array}\right]$$

(41) $$\Gamma_{b}=\left[\begin{array}{ccccccc} C_{b} & 0_{N_{y1}\times \left(N_{x}+N_{u}\right)} & 0_{N_{y1}\times \left(N_{x}+N_{u}\right)} & \cdots & 0_{N_{y1}\times \left(N_{x}+N_{u}\right)} & \cdots & 0_{N_{y1}\times \left(N_{x}+N_{u}\right)}\\ C_{b}A & C_{b} & 0_{N_{y1}\times \left(N_{x}+N_{u}\right)} & \cdots & 0_{N_{y1}\times \left(N_{x}+N_{u}\right)} & \cdots & 0_{N_{y1}\times \left(N_{x}+N_{u}\right)}\\ C_{b}A^{2} & C_{b}A & C_{b} & \cdots & 0_{N_{y1}\times \left(N_{x}+N_{u}\right)} & \cdots & 0_{N_{y1}\times \left(N_{x}+N_{u}\right)}\\ \vdots & \vdots & \vdots & \vdots & \vdots & \vdots & \vdots \\ C_{b}A^{c-1} & C_{b}A^{c-2} & C_{b}A^{c-3} & \cdots & C_{b} & \cdots & 0_{N_{y1}\times \left(N_{x}+N_{u}\right)}\\ \vdots & \vdots & \vdots & \vdots & \vdots & \vdots & \vdots \\ C_{b}A^{p-1} & C_{b}A^{p-2} & C_{b}A^{p-3} & \cdots & C_{b}A^{p-c} & \cdots & C_{b} \end{array}\right]$$

Based on the road adhesion conditions and the tire slip angle limits, it can be shown in Equation (42).

(42) $$Y_{b,\min}\leq \Phi_{b}\xi \left(k\right)+\Theta_{b}\Delta U\left(k\right)+\Gamma_{b}D\left(k\right)\leq Y_{b,\max}$$

In summary, the finite time domain optimization problem for MPC in this paper can be transformed into a standard quadratic programming problem:

(43) $$\begin{array}{l} \underset{\Delta U\left(k\right)}{\min}\Delta U{\left(k\right)}^{T}\left(\Theta^{T}Q\Theta +R\right)\Delta U\left(k\right)+2E^{T}Q\Theta \Delta U\left(k\right)\\ \begin{array}{ll} s.t. & \Delta U_{\min}\left(k\right)\leq \Delta U\left(k\right)\leq \Delta U_{\max}\left(k\right)\\ & U_{\min}\left(k\right)\leq U_{ct}\left(k-1\right)+A_{ct}*\Delta U\left(k\right)\leq U_{\max}\left(k\right)\\ & Y_{b,\min}\leq \Phi_{b}\xi \left(k\right)+\Theta_{b}\Delta U\left(k\right)+\Gamma_{b}D\left(k\right)\leq Y_{b,\max} \end{array} \end{array}$$

The optimal sequence of control increments is obtained by solving the standard quadratic programming problem, and the first element $\Delta u^{*}\left(k\right)$ is output. The control variable at the current moment can be described as Equation (44).

(44) $$u\left(k\right)=u\left(k-1\right)+\Delta u^{*}\left(k\right)$$

### 2.5. Coordinated Control

The coordinated control layer receives the generalized force, generalized yaw moment, and generalized wheel angle from the upper layer. The wheel torque and the wheel angle are calculated and output to in-wheel motors and steering motors. Depending on the actuating subsystem, the coordinated control layer consists of two parts: torque distribution control and wheel angle distribution control.

#### 2.5.1. Torque Distribution Control

The torque distribution control receives the generalized longitudinal force and the generalized yaw moment output from the upper level and distributes and controls the driving torque of the individual wheels. The current research in torque distribution mainly considers the stability index and economic index as the control objective and uses online real-time optimization to complete the torque coordinated distribution [33,34]. However, current research has the following limitations:

- The rule-based torque distribution control does not consider the operating efficiency of motor, resulting in unnecessary power loss.
- The real-time optimization of torque distribution places a large burden on the controller. The solution speed may be slow and this problem may even be unsolvable under certain working conditions.
- The economic evaluation generally uses the size of the control variables as the index, ignoring the efficiency characteristics of the motor. This is mainly due to the nonlinearity of the motor model, which makes it impossible to optimize the system efficiency in real-time.

To address these issues, this paper simplifies the real-time optimization problem of torque distribution in order to balance practical and optimization objectives. A combination of rule-based strategy and offline optimization strategy is used for the distribution strategy. A rule-based strategy is designed to distribute the torque between the left and right wheels. Considering the stability constraints, the optimization of the torque distribution coefficients between the front and rear wheels based on the MPSO algorithm leads to an optimal wheel torque, improving the efficiency while ensuring the response speed of the vehicle. First, the torque distribution between left and right wheels is carried out based on the generalized longitudinal force and generalized yaw moment requirements. Then, the MPSO algorithm is used to optimize the torque distribution offline between the front and rear wheels on the same side to obtain the optimal distribution coefficient of the front axle. Finally, the driving torque of each wheel is calculated.

1. Left-right distribution

A rule-based strategy is used for the generalized longitudinal force to distribute it equally between the left and right sides of the vehicle. The corresponding demand torques are obtained as follows.

(45) $$T_{dlF}=T_{drF}=\frac{F_{xd}}{2}R_{eff}$$

where, $T_{dlF}$ and $T_{drF}$ are the left-hand and right-hand demand torques, respectively, corresponding to the generalized longitudinal forces.

The same uniform distribution is used for the generalized yaw moment. The yaw moment generated by the wheels on both sides is equal and has an opposite direction, which can shorten the response time of the vehicle and speed up the response speed. It can be represented as Equation (46).

(46) $$\begin{array}{l} T_{dlM}=-\frac{M_{zd}}{d}R_{eff}\\ T_{drM}=\frac{M_{zd}}{d}R_{eff} \end{array}$$

where, $T_{dlM}$ and $T_{drM}$ are the left-hand and right-hand demand torques, respectively corresponding to the generalized yaw moment.

The total demand torques are,

(47) $$\begin{array}{l} T_{dl}=\frac{F_{xd}}{2}R_{eff}-\frac{M_{zd}}{d}R_{eff}\\ T_{dr}=\frac{F_{xd}}{2}R_{eff}+\frac{M_{zd}}{d}R_{eff} \end{array}$$

where, $T_{dl}$ and $T_{dr}$ are the left-hand and right-hand demand torques, respectively.

2. Front-rear distribution

For the front and rear wheels on the same side, the total demand torque is fixed. The independent drive function allows the torque of the front and rear wheels to be optimally distributed so that in-wheel motors can operate in the high-efficiency range as far as possible. Considering the high time complexity of online optimization, this paper uses the MPSO algorithm to optimize the distribution coefficient of the front axle offline, and it is put into a tabular form for subsequent table lookup operations which ensures the real-time performance of the system.

(1) The MPSO algorithm

PSO (Particle Swarm Optimization) is a type of swarm intelligent optimization algorithm [35]. In PSO, each solution of the optimization problem is abstracted as a particle, and all particles search for the optimal solution in the solution space. Each particle is assigned a fitness function to determine the fitness of the current location, and a speed property to determine the distance and direction of flight, after which the optimal solution is determined by iteration.

**Figure 5:**
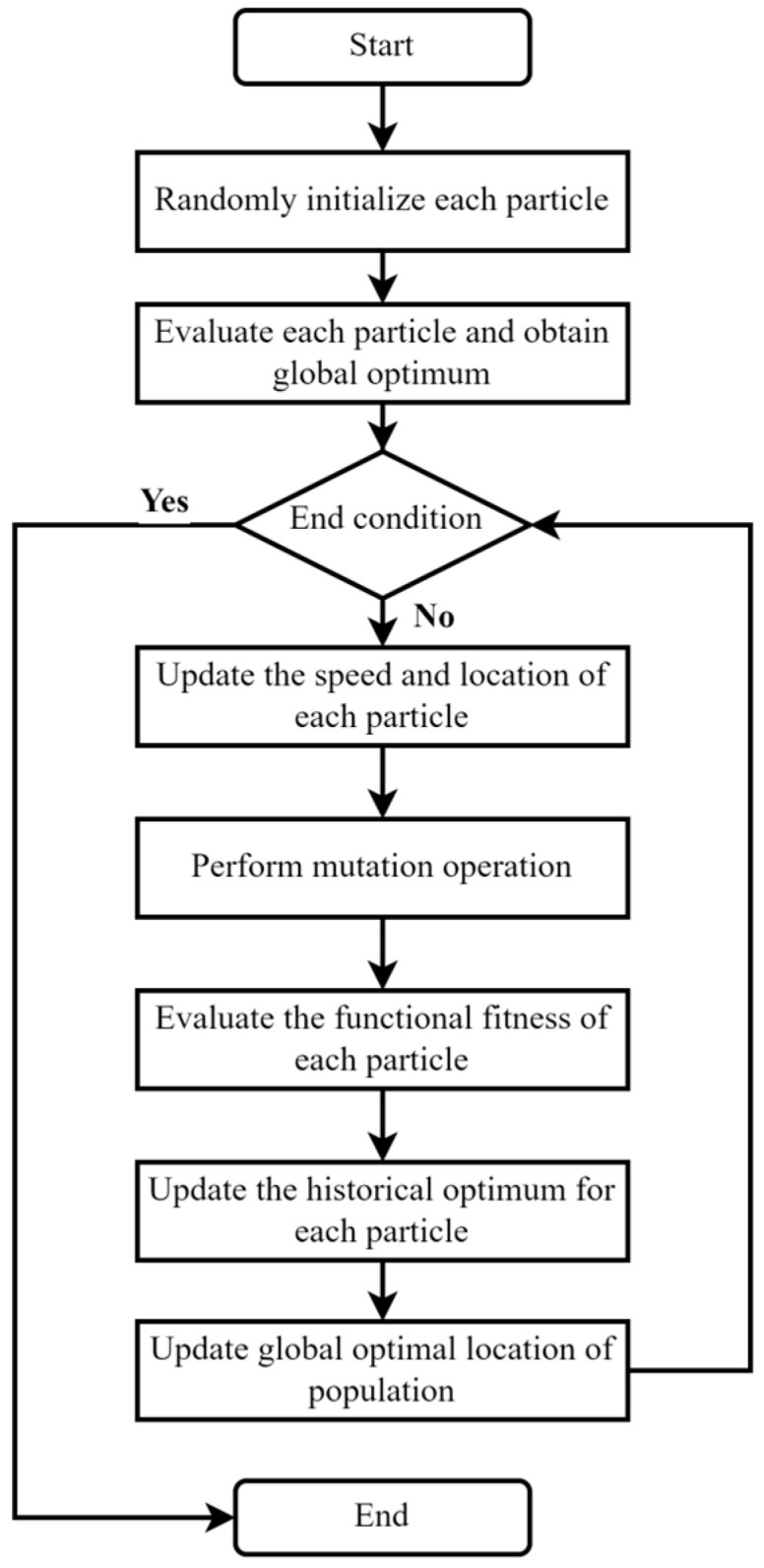
Flowchart of MPSO algorithm.

The traditional PSO algorithm has the advantage of fast convergence, but it can easily fall into local optimal solutions in some complex situations. Therefore, the MPSO algorithm is introduced to avoid the problem of premature convergence. The MPSO algorithm combines the traditional PSO algorithm with the idea of mutation in the genetic algorithm. The mutation occurs when the population location is updated, thereby jumping out of the local optimal solution, which is conducive to finding the global optimal solution and reduces the probability of premature convergence. The process of the MPSO is shown in Figure 5.

(2) Distribution coefficient optimization

Since the four in-wheel motors are the same, the offline optimization problems on the left and right sides are essentially the same, so this paper takes the right-side wheel as an example. In order to carry out the optimization, the economy needs to be characterized and this paper uses the overall motor efficiency as the economic index. The expression of the overall motor efficiency is,

(48) $$J=\frac{T_{fr}n_{fr}+T_{rr}n_{rr}}{\frac{T_{fr}n_{fr}}{\eta_{fr}}+\frac{T_{rr}n_{rr}}{\eta_{rr}}}$$

where, $T_{fr}$ and $T_{rr}$ are the output torques of the right front and right rear motors, respectively, $n_{fr}$ and $n_{rr}$ are the output speeds of the right front and right rear motors, respectively, and $\eta_{fr}$ and $\eta_{rr}$ are the output efficiencies of the right front and right rear motors, respectively.

To simplify the complexity of the optimization problem, the concept of the front axle distribution coefficient λ is introduced, which is the ratio of the front wheel torque to the total demand torque.

During normal driving, there is little difference in speed between the front and rear wheels on the same side, so the optimization problem can be translated into Equation (49).

(49) $$\min J=\frac{\lambda }{\eta_{fr}}+\frac{1-\lambda }{\eta_{rr}}$$

Considering the constraints on the external characteristics of the in-wheel motor, the following constraints are made:

(50) $$\begin{array}{l} 0\leq \lambda T_{dr}\leq T_{\max}\\ 0\leq \left(1-\lambda \right)T_{dr}\leq T_{\max} \end{array}$$

where, $T_{\max}$ is the peak torque of the in-wheel motor.

In addition, depending on the vehicle model, the center of mass is closer to the front axle, which means that the vertical load on the front axle is greater than that on the rear axle. This results in a greater tire adhesion limit for the front wheel, with a greater longitudinal force limit. Therefore, the front wheels should take on a larger portion of the required torque. The following restriction applies to the distribution coefficient of the front axle.

(51) 0.5≤λ≤1

**Figure 6:**
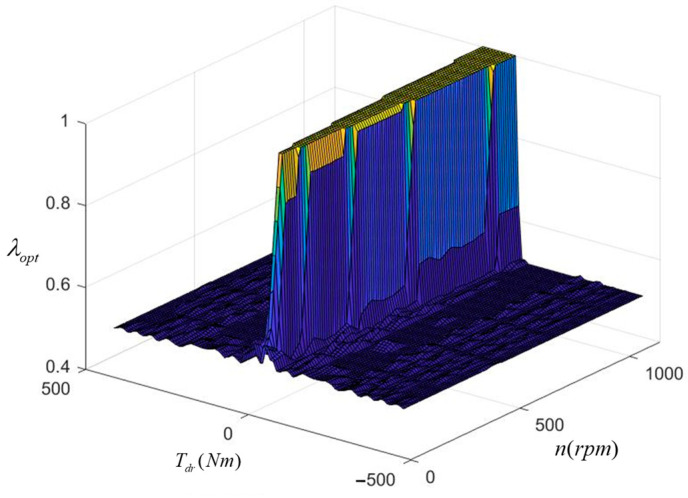
Optimal front axle distribution coefficient.

Factors affecting the front axle distribution coefficient include the velocity and the demand torque, wherein the velocity is represented by wheel speed. Based on the MPSO algorithm, the offline optimization of the front axle distribution coefficient is realized by programming in MATLAB. Finally, the mapping of the optimal distribution coefficient for the front axle is shown in Figure 6. The optimal distribution coefficient for the front axle is determined by the velocity and the demand torque, which realizes the torque distribution between the front and rear wheels on the same side. From the optimization results, when the demand torque is low, the front wheel drive is selected; when the demand torque is high, it tends to be four-wheel drive.

(3) Torque calculation for each wheel

Based on the optimal coefficient for the distribution of front axle torque determined via offline optimization, the torque for each wheel can be calculated. The offline optimization of the front axle distribution coefficients takes into account the constraints on the peak torque of the in-wheel motors but still requires stability corrections according to the constraints on road adhesion, as shown in Equation (52).

(52) $$\left\{\begin{array}{l} T_{fl}=\min\left(\left|\lambda_{l}T_{dl}\right|,\mu F_{zfl}\right)\cdot \mathrm{sgn}\left(T_{dl}\right)\\ T_{fr}=\min\left(\left|\lambda_{r}T_{dr}\right|,\mu F_{zfr}\right)\cdot \mathrm{sgn}\left(T_{dr}\right)\\ T_{rl}=\min\left(\left|\left(1-\lambda_{l}\right)T_{dl}\right|,\mu F_{zrl}\right)\cdot \mathrm{sgn}\left(T_{dl}\right)\\ T_{rr}=\min\left(\left|\left(1-\lambda_{r}\right)T_{dr}\right|,\mu F_{zrr}\right)\cdot \mathrm{sgn}\left(T_{dr}\right) \end{array}\right.$$

where, $\lambda_{l}$ and $\lambda_{r}$ are the optimal front axle distribution coefficients for the left and right side, respectively. $T_{ij}$ is the torque of each wheel, μ is the coefficient of road adhesion, and $F_{zij}$ is the vertical load of each wheel, which can be obtained using Equation (53).

(53) $$\left\{\begin{array}{c} F_{zfl}=\frac{mgl_{r}}{2l}-\frac{ma_{x}h}{2l}-\frac{ma_{y}hl_{r}}{dl}\\ F_{zfr}=\frac{mgl_{r}}{2l}-\frac{ma_{x}h}{2l}+\frac{ma_{y}hl_{r}}{dl}\\ F_{zrl}=\frac{mgl_{f}}{2l}+\frac{ma_{x}h}{2l}-\frac{ma_{y}hl_{f}}{dl}\\ F_{zrr}=\frac{mgl_{f}}{2l}+\frac{ma_{x}h}{2l}+\frac{ma_{y}hl_{f}}{dl} \end{array}\right.$$

#### 2.5.2. Wheel Angle Distribution Control

The generalized wheel angle calculated by the target planning layer is the corresponding angle at the center of the front and rear axles, which is referred to as the equivalent angle of the front and rear wheels. Traditional control methods often assume that the left and right wheels on the same axis have the same angle, which directly corresponds to the equivalent angle. Although this method can simplify the design process, it does not take into account the actual steering geometry relationship, which not only increases tire wear but also causes unnecessary energy consumption. For this reason, the wheel angle distribution method based on Ackermann steering geometry is applied in this paper to convert the equivalent angle into the angle of each wheel.

By distributing the wheel angles based on the Ackermann steering geometry, the tires can be put into a pure rolling condition as much as possible. However, the ideal Ackermann steering principle is limited by the fact that it ignores the cornering characteristics of the tires. To further improve vehicle stability and reduce excessive tire wear, the Ackermann principle must be modified to obtain wheel angles.

When the vehicle is steering, the lateral elastic deformation of the tires results in the slip angles of the individual wheels on the front and rear axles. As shown in Equation (54), the corresponding relationship that each wheel angle should satisfy can be called the modified Ackermann principle.

(54) $$\left\{\begin{array}{l} \tan(\delta_{fl}-\alpha_{fl})=\frac{\tan\delta_{f}}{1-\frac{d}{2l}(\tan\delta_{f}-\tan\delta_{r})}\\ \tan(\delta_{fr}-\alpha_{fr})=\frac{\tan\delta_{f}}{1+\frac{d}{2l}(\tan\delta_{f}-\tan\delta_{r})}\\ \tan(\delta_{rl}-\alpha_{rl})=\frac{\tan\delta_{r}}{1-\frac{d}{2l}(\tan\delta_{f}-\tan\delta_{r})}\\ \tan(\delta_{rr}-\alpha_{rr})=\frac{\tan\delta_{r}}{1+\frac{d}{2l}(\tan\delta_{f}-\tan\delta_{r})} \end{array}\right.$$

## 3. Simulation and Results

This section is divided into subheadings. It should provide a concise and precise description of the experimental results, their interpretation, as well as the experimental conclusions that can be drawn.

### 3.1. Environment and Configuration

**Figure 7:**
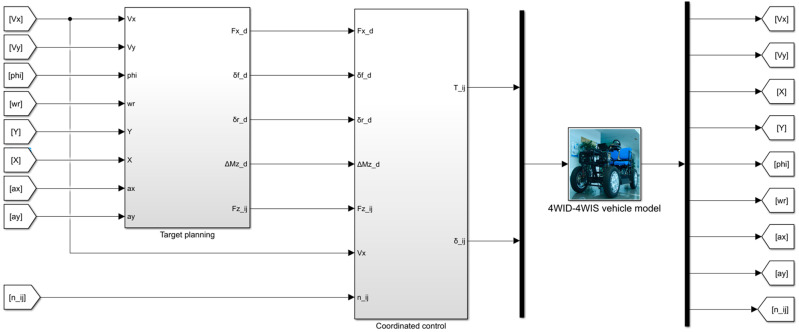
4WID-4WIS EV simulation model.

In this section, the simulation model is built based on the MATLAB R2022a software produced by MathWorks (Natick, MA, USA), as shown in Figure 7. The vehicle model is the seven degrees-of-freedom vehicle model presented in Section 2. The simulations were performed using single-lane change and slalom test. The results verify the effect of trajectory tracking and economic optimization, which fully proves the effectiveness of the proposed control strategy.

To validate the economic performances of the coordinated control, simulations comparing the average distribution strategy and the wheel load distribution strategy are performed. These two strategies are simple and efficient and are widely used in practice. Therefore, the advantages of the proposed distribution control strategy can be further emphasized.

#### 3.1.1. Average Distribution Strategy

The generalized longitudinal force and the generalized yaw moment output from the upper layer are distributed equally to each wheel. The wheel torque in this strategy is as follows:

(55) $$\left\{\begin{array}{c} T_{fl}=T_{rl}=\frac{F_{xd}}{4}R_{eff}-\frac{M_{zd}}{2d}R_{eff}\\ T_{fr}=T_{rr}=\frac{F_{xd}}{4}R_{eff}+\frac{M_{zd}}{2d}R_{eff} \end{array}\right.$$

#### 3.1.2. Wheel Load Distribution Strategy

The generalized force and yaw moment are distributed equally between the left and right sides of the vehicle. Then, the distribution between the front and rear wheels is carried out on the same side in proportion to the vertical load on the tires. The wheel torque with this strategy is shown in Equation (56).

(56) $$\left\{\begin{array}{c} T_{fl}=\frac{F_{zfl}}{F_{zfl}+F_{zrl}}\left(\frac{F_{xd}}{2}R_{eff}-\frac{M_{zd}}{d}R_{eff}\right)\\ T_{fr}=\frac{F_{zfr}}{F_{zfr}+F_{zrr}}\left(\frac{F_{xd}}{2}R_{eff}+\frac{M_{zd}}{d}R_{eff}\right)\\ T_{rl}=\frac{F_{zrl}}{F_{zfl}+F_{zrl}}\left(\frac{F_{xd}}{2}R_{eff}-\frac{M_{zd}}{d}R_{eff}\right)\\ T_{rr}=\frac{F_{zrr}}{F_{zfr}+F_{zrr}}\left(\frac{F_{xd}}{2}R_{eff}+\frac{M_{zd}}{d}R_{eff}\right) \end{array}\right.$$

### 3.2. Results and Analysis

#### 3.2.1. Single-Lane Change

As one of the common conditions, the single-lane change condition is usually used to simulate a vehicle lane change scenario.

1. Trajectory tracking effect

**Figure 8:**
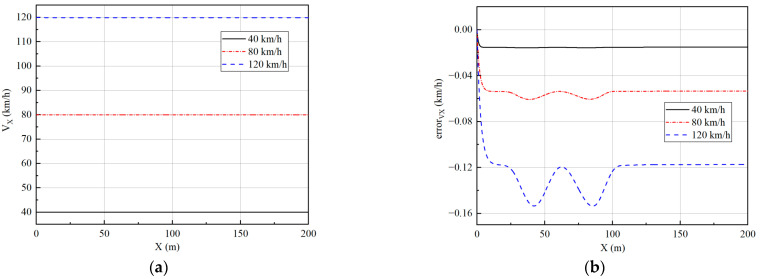
Velocity tracking results under single-lane change: (**a**) The results of velocity; (**b**) The results of longitudinal tracking error.

**Figure 9:**
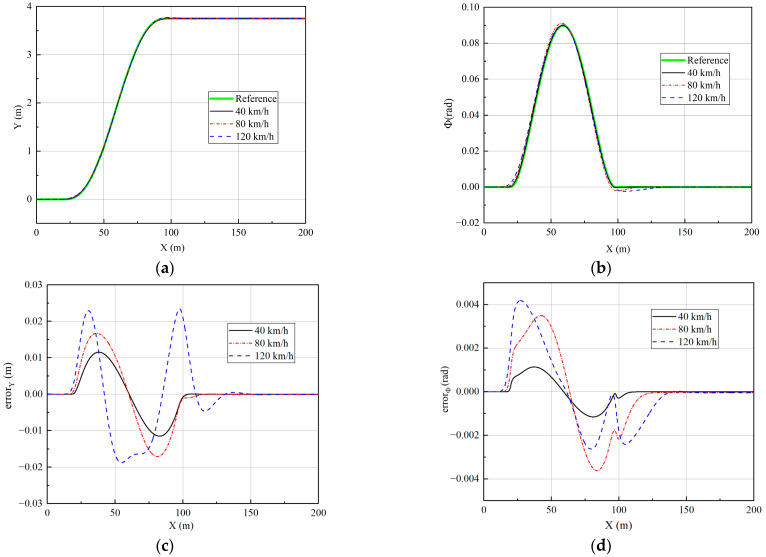
Path tracking results under single-lane change: (**a**) The results of lateral displacement; (**b**) The results of heading angle; (**c**) The results of lateral displacement tracking error; (**d**) The results of heading angle tracking error.

Three speeds of 40 km/h, 80 km/h, and 120 km/h are simulated to verify the effect of the controller. The longitudinal velocity tracking and lateral path tracking results are analyzed to verify the performance of the vehicle at low, medium, and high speeds. The simulation results are shown in Figure 8 and Figure 9.

For a single-lane change, the longitudinal velocity tracking algorithm can track the expected velocity very well according to Figure 8. The tracking error is less than 0.2 km/h, which satisfies the longitudinal tracking accuracy requirements. As the velocity increases, the tracking error gradually increases. This is mainly because the nonlinearity of the tire gradually increases and is associated with the strong coupling properties between the subsystems, resulting in a decrease in tracking accuracy.

The data in Figure 9c,d have been processed to obtain the tracking error at different velocities to facilitate quantitative analysis of the error, as shown in Table 1.

From Figure 9 and Table 1, it can be seen that the lateral path tracking algorithm is very good at tracking the desired path for single-lane change. As the speed increases, the lateral displacement error gradually increases. The maximum lateral displacement error is 0.0115 m at 40 km/h, 0.0171 m at 80 km/h, and 0.0234 m at 120 km/h, which is mainly due to the gradual increase in the nonlinearity of the tire and the enhancement of the coupling effect of the subsystem. At the same time, although the lateral displacement error increases, it is still generally small and within acceptable limits. Similarly, the heading angle error also increases with velocity but remains at a low level overall. The maximum heading angle error at 120 km/h is only 0.0042 rad, which demonstrates the good path-tracking performance of the controller.

The trajectory tracking controller is designed to achieve good stability while providing accurate and reliable path tracking. To check the stability effect, the lateral acceleration of the vehicle, side slip angle, and yaw rate are selected as indicators, and the simulation results are analyzed.

**Figure 10:**
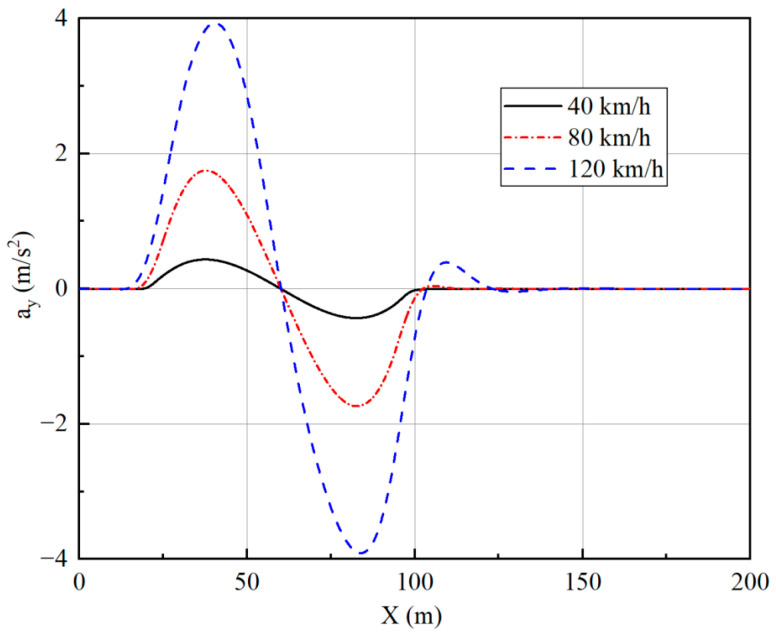
Lateral acceleration under single-lane change.

**Figure 11:**
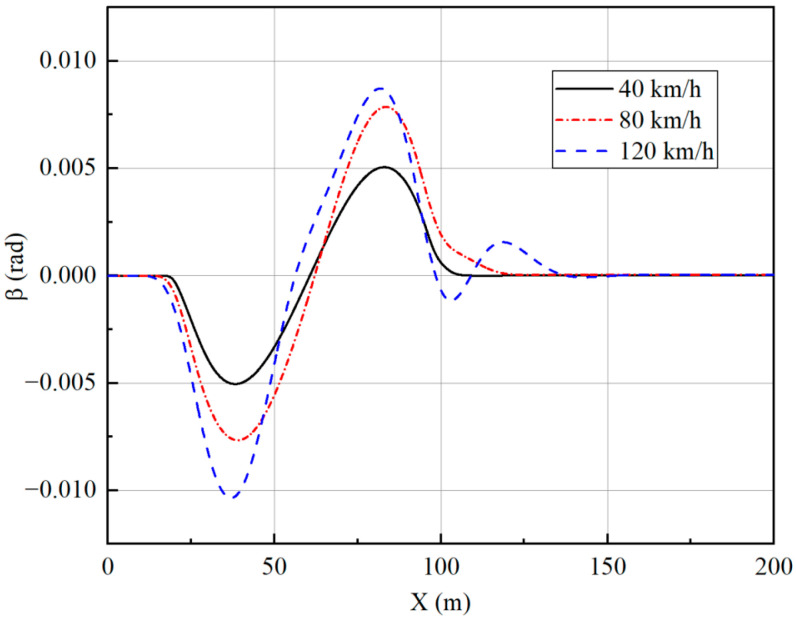
Side slip angle under single-lane change.

**Figure 12:**
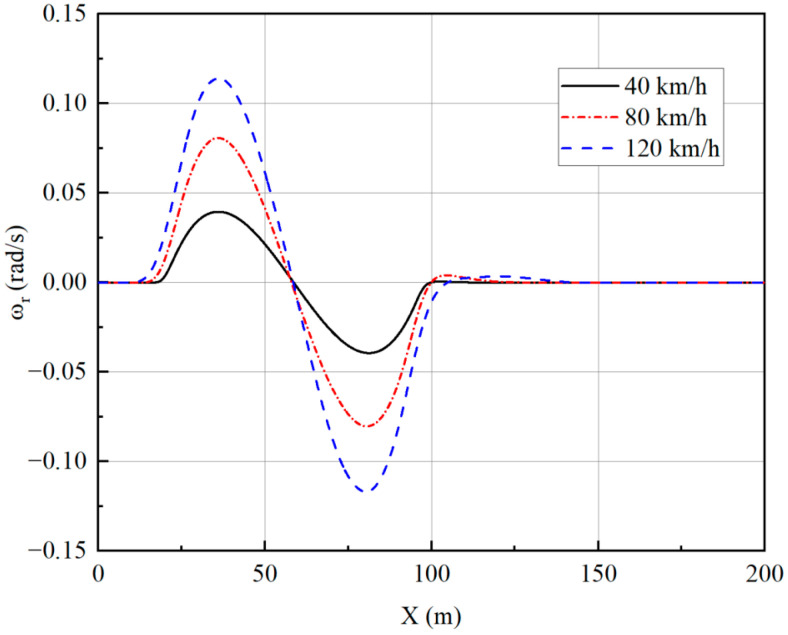
Yaw rate under single-lane change.

**Figure 13:**
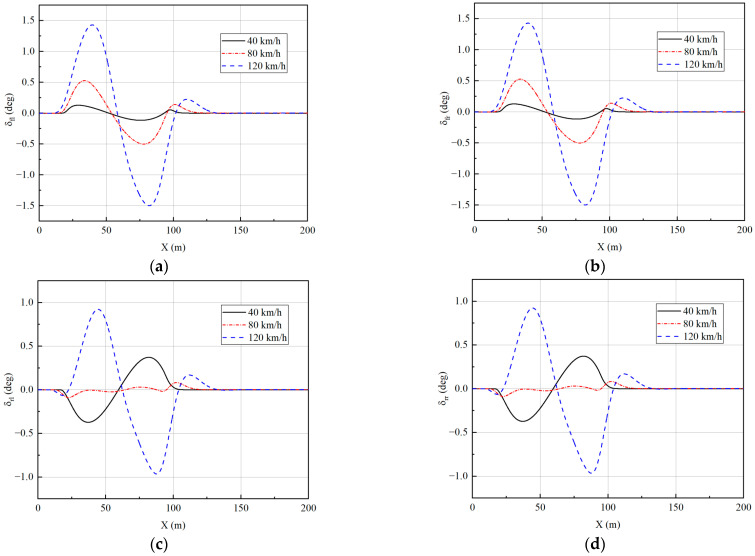
Wheel angles under single-lane change: (**a**) Front-left wheel; (**b**) Front-right wheel; (**c**) Rear-left wheel; (**d**) Rear-right wheel.

From Figure 10, Figure 11, Figure 12 and Figure 13, it can be seen that the vehicle is in a stable state due to the stability constraints and no instability occurs even at a high speed of 120 km/h. The lateral acceleration is kept within 0.4 g, which ensures the accuracy of the linear tire model. The side slip angles at different speeds are all within 0.01 rad, and the yaw rate is all within 0.15 rad/s, indicating that the vehicle has good driving stability.

The simulation results shown in Figure 13 illustrate the advantages of 4WIS in terms of dynamic control. At low speeds, the front and rear wheels rotate in the opposite direction, reducing the steering radius and improving the vehicle’s maneuverability. At high speeds, the front and rear wheels turn in the same direction, thereby increasing the lateral stability margin and improving the vehicle’s driving stability.

2. Economy optimization effect

**Figure 14:**
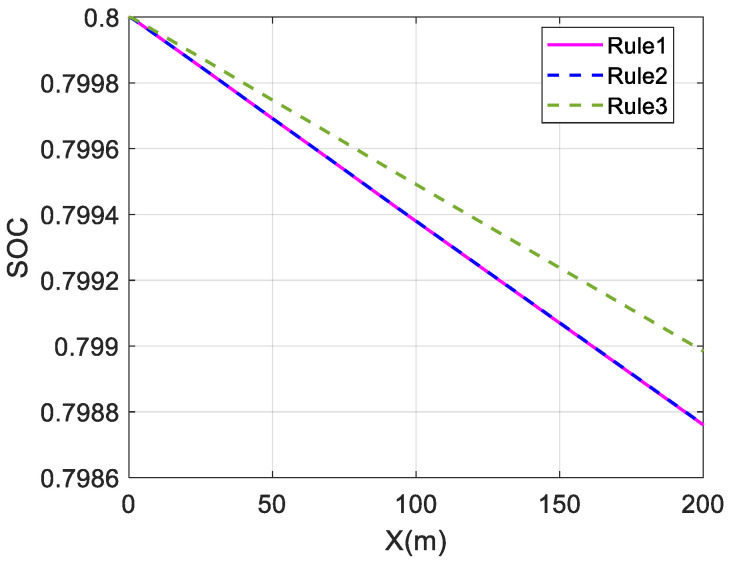
Change in SOC at 40 km/h under single-lane change.

**Figure 15:**
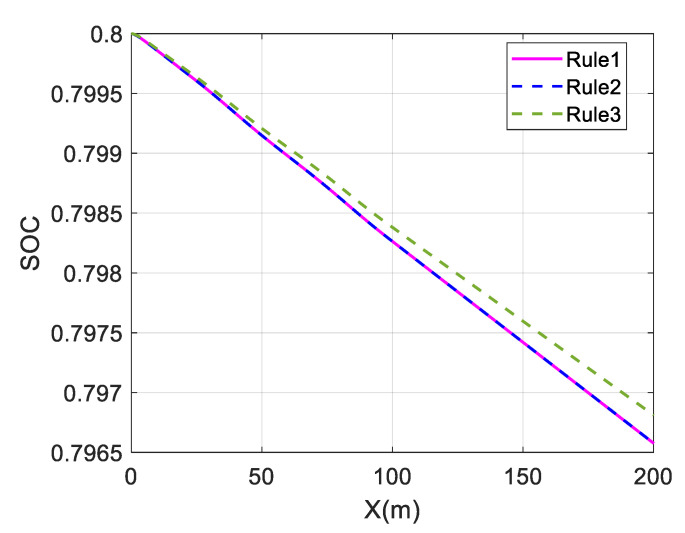
Change in SOC at 80 km/h under single-lane change.

**Figure 16:**
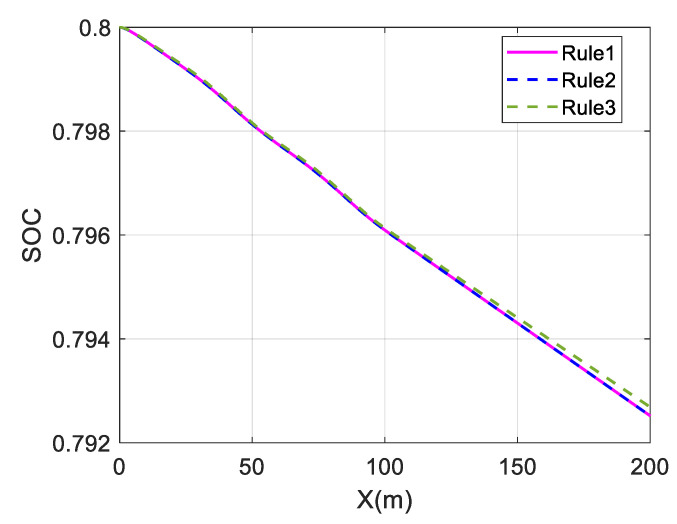
Change in SOC at 80 km/h under single-lane change.

The above three distribution strategies are simulated at different velocities to validate the economy optimization effect, using the overall efficiency of the motor and the battery SOC as performance indicators. It is assumed that Rule 1 is the average distribution strategy, Rule 2 is the wheel load distribution strategy, and Rule 3 is the torque distribution strategy proposed in this paper. At the same time, the control strategy does not include a braking energy recovery strategy. The initial value of SOC is set to 0.8. The simulation results are shown in Figure 14, Figure 15 and Figure 16 and Table 2.

For a single-lane change, the change in SOC is almost identical for the average and wheel load distribution strategies at different speeds. The maximum and average values of the overall motor efficiency are also almost identical, indicating that the economic effects of these two strategies are almost the same.

The change in battery SOC represents the electrical energy consumed by the in-wheel motors while driving. Comparing the change curves of SOC at different speeds, the SOC change in Rule 3 is smaller than those in Rule 1 and Rule 2, and the SOC in Rule 3 falls relatively slowly. This shows that the in-wheel motors consume less electric energy in the Rule 3 strategy, which proves the energy economy of the proposed strategy.

The average value of the overall motor efficiency of Rule 3 is 14.68% higher than that of Rule 1 at 40 km/h. Compared with the results of Rule 1, the average values at 80 km/h and 120 km/h have increased by 4.10% and 3.5%, respectively. This shows that the coordinated control strategy proposed in this paper can maintain the overall motor efficiency at a relatively high level and effectively improve the vehicle economy.

Comparing the effects of economy optimization at different speeds, it can be found that the economy optimization at low speeds is better, and the difference in SOC decline is significant. This is because the demand torque is relatively low at low speeds. In the average distribution strategy, the torque of each wheel is very low, so in-wheel motors operate in the low-efficiency region. However, the proposed strategy optimizes the torque distribution and tends to select the front wheel drive mode. This allows the front in-wheel motors to operate in a relatively efficient range, and the overall efficiency can be significantly improved. However, at high speeds, the in-wheel motors’ speeds are also high. As shown in Figure 2, the motor’s efficiency characteristic range gradually narrows as the speed increases when the motor’s speed is high. The distribution difference of the motor working points decreases for different strategies, resulting in a less significant improvement effect.

#### 3.2.2. Slalom Test

1. Trajectory tracking effect

**Figure 17:**
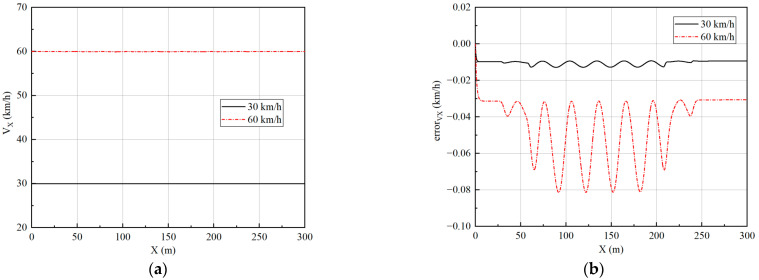
Velocity tracking results under slalom test: (**a**) The results of velocity; (**b**) The results of longitudinal tracking error.

**Figure 18:**
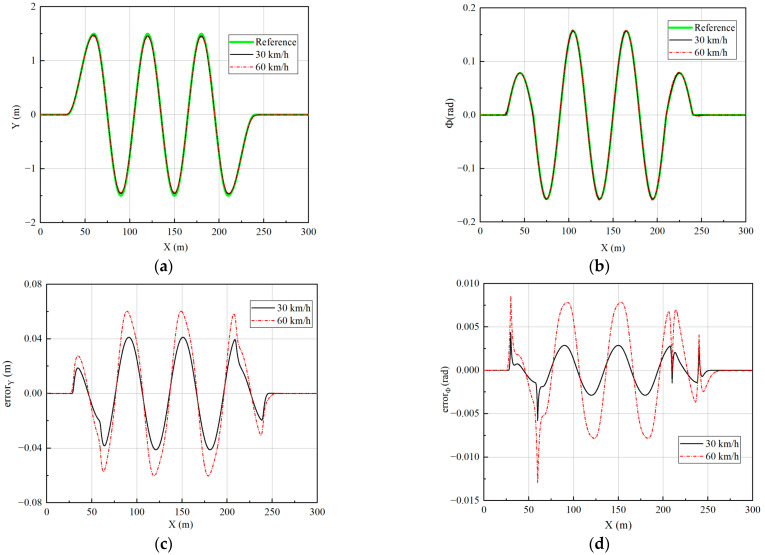
Path tracking results under slalom test: (**a**) The results of lateral displacement; (**b**) The results of heading angle; (**c**) The results of lateral displacement tracking error; (**d**) The results of heading angle tracking error.

Two speeds of 30 km/h and 60 km/h are simulated to verify the effect of the controller. The longitudinal velocity tracking and lateral path tracking results are analyzed to verify the performance of the vehicle at different speeds. The simulation results are shown in Figure 17 and Figure 18.

The data in Figure 18c,d have been processed to obtain the tracking error at different velocities to facilitate quantitative analysis of the error, as shown in Table 3.

From Figure 18 and Table 3, it can be seen that the lateral path tracking algorithm is very good at tracking the desired path in a slalom test. As the speed increases, the path-tracking error gradually increases. The average lateral displacement error is 0.0158 m and the average heading angle error is 0.0011 rad at 30 km/h, while the average lateral displacement error is 0.0241 m and the average heading angle error is 0.0033 rad at 60 km/h. Although the tracking error increases, it remains low overall, which shows the good tracking performance of the designed controller.

**Figure 19:**
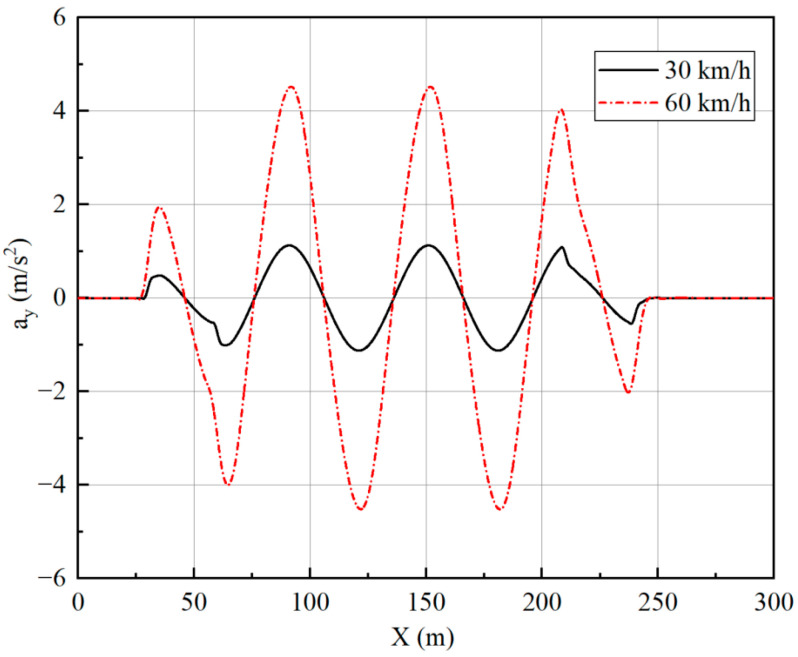
Lateral acceleration under slalom test.

**Figure 20:**
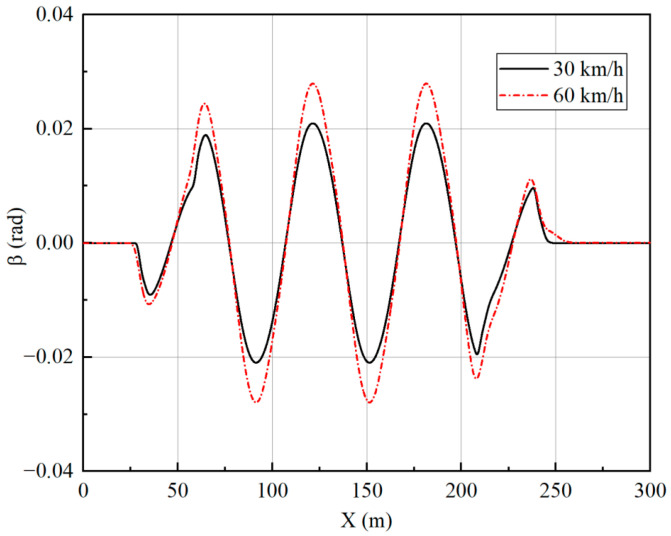
Side slip angle under slalom test.

**Figure 21:**
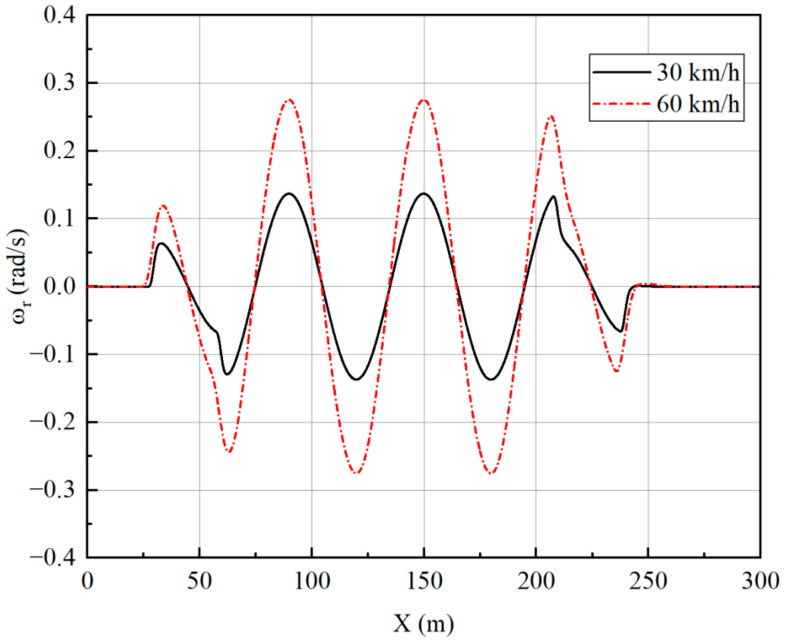
Yaw rate under slalom test.

**Figure 22:**
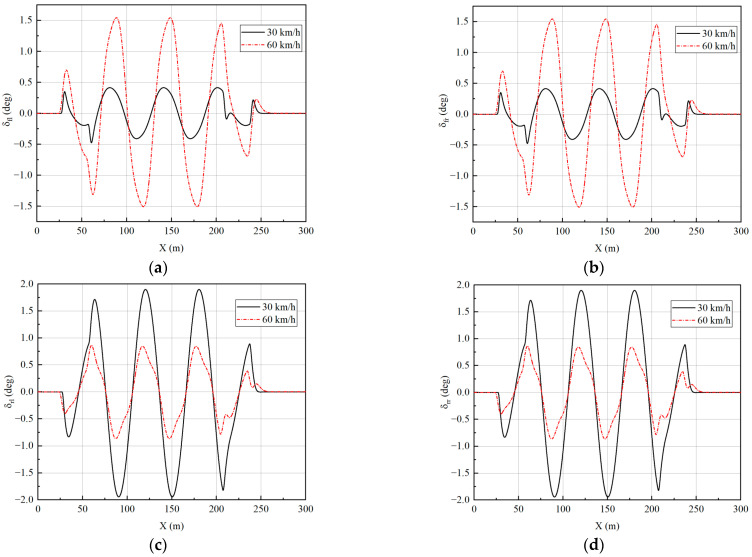
Wheel angles under slalom test: (**a**) Front-left wheel; (**b**) Front-right wheel; (**c**) Rear-left wheel; (**d**) Rear-right wheel.

Figure 19, Figure 20, Figure 21 and Figure 22 show that the vehicle is in a stable state due to stability constraints. The side slip angle is maintained at a low level, which provides good tracking capability. The lateral acceleration is kept within 0.4 g, ensuring the accuracy of the linear tire model. The velocities under both simulation conditions are not particularly high, so the front and rear wheels turn in opposite directions, which improves the maneuverability of the vehicle while meeting the stability requirements.

2. Economy optimization effect

**Figure 23:**
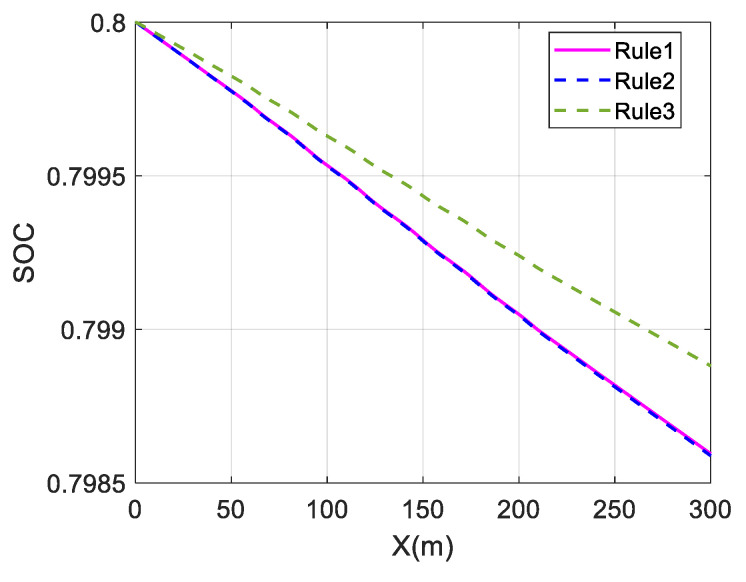
Change in SOC at 30 km/h under slalom test.

**Figure 24:**
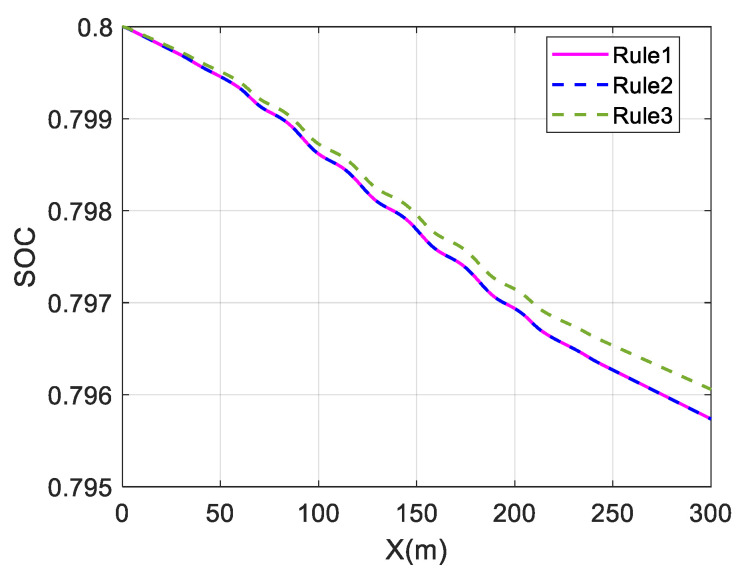
Change in SOC at 80 km/h under slalom test.

The above three distribution strategies are simulated at different speeds to validate the economy optimization effect, using the overall efficiency of the motor and the battery SOC as performance indicators. The simulation results are shown in Figure 23 and Figure 24 and Table 4.

In the slalom test, the change in SOC is almost identical for the average and wheel load distribution strategies at different speeds. The maximum and average values of the overall motor efficiency are also almost identical, indicating that the economic effects of these two strategies are almost the same.

Comparing the change curves of SOC at different speeds, the change in SOC in Rule 3 is smaller than in Rule 1 and Rule 2, and the SOC in Rule 3 drops relatively slowly. This shows that the in-wheel motors consume electric energy under the Rule 3 strategy, which proves the energy economy of the proposed strategy.

The coordinated control proposed in this paper is significantly better than the other two strategies, as shown in Table 4. Compared with the results of Rule 1, the average value of the overall motor efficiency of Rule 3 has increased by 21.67% at 30 km/h. At 60 km/h, it has increased by 10.43%, which proves that the coordinated control strategy can effectively improve the vehicle economy.

## 4. Conclusions

In this paper, a 4WID-4WIS EV trajectory tracking coordinated control strategy considering energy consumption economy is proposed to improve vehicle stability and economy during trajectory tracking. A hierarchical chassis coordinated control architecture for 4WID-4WIS EV is designed. In the target planning layer, the longitudinal velocity tracking and lateral path tracking are achieved by using expert PID and MPC, respectively, considering stability constraints, such as yaw rate and tire slip angle. In the coordinated control layer, the optimal torque distribution for each wheel is performed to achieve the optimal overall motor efficiency based on the MPSO algorithm. Then, the angle distribution of each wheel is performed in combination with the modified Ackermann theory. From the simulation results, it can be concluded that the proposed coordinated control strategy not only achieves good trajectory tracking but also ensures driving stability and improves the energy consumption economy.

In future work, the coupling characteristics between multiple subsystems will be considered. The influence of vertical dynamics will be incorporated into the control strategy to achieve unified control of multi-dimensional lateral, longitudinal, and vertical dynamics. At the same time, the motor model will be further improved, and research on redundant control of actuator failures will be conducted. The test with a real vehicle will be conducted to further verify the effectiveness of the control strategy.

## Figures and Tables

**Table 1:** Comparison of path tracking errors under single-lane change.

	Performance Index	40 km/h	80 km/h	120 km/h
Lateral displacement tracking error (m)	Maximum	0.0115	0.0171	0.0234
	Average	0.0024	0.0036	0.0053
	Standard deviations	0.0040	0.0058	0.0075
Heading angle tracking error (rad)	Maximum	0.0012	0.0036	0.0042
	Average	0.0002	0.0009	0.0009
	Standard deviations	0.0004	0.0012	0.0012

**Table 2:** Comparison of overall motor efficiency under single-lane change.

Velocity (km/h)	Strategy	Maximum	Average
40	Rule 1	0.7356	0.7303
	Rule 2	0.7347	0.7283
	Rule 3	0.8411	0.8375
80	Rule 1	0.8969	0.8877
	Rule 2	0.8970	0.8882
	Rule 3	0.9276	0.9241
120	Rule 1	0.8973	0.8791
	Rule 2	0.8972	0.8793
	Rule 3	0.9147	0.9107

**Table 3:** Comparison of path tracking errors under slalom test.

	Performance Index	30 km/h	60 km/h
Lateral displacement tracking error (m)	Maximum	0.0412	0.0603
	Average	0.0158	0.0241
	Standard deviations	0.0147	0.0214
Heading angle tracking error (rad)	Maximum	0.0058	0.0129
	Average	0.0011	0.0033
	Standard deviations	0.0010	0.0029

**Table 4:** Comparison of overall motor efficiency under slalom test.

Velocity (km/h)	Strategy	Maximum	Average
30	Rule 1	0.6404	0.6151
	Rule 2	0.6409	0.6121
	Rule 3	0.7857	0.7484
60	Rule 1	0.8973	0.7995
	Rule 2	0.8971	0.7987
	Rule 3	0.9345	0.8829

## Data Availability

Not applicable.
